# Comparative transcriptomics provides novel insights into the mechanisms of selenium tolerance in the hyperaccumulator plant *Cardamine hupingshanensis*

**DOI:** 10.1038/s41598-018-21268-2

**Published:** 2018-02-12

**Authors:** Yifeng Zhou, Qiaoyu Tang, Meiru Wu, Di Mou, Hui Liu, Shouchuang Wang, Chi Zhang, Li Ding, Jie Luo

**Affiliations:** 1 National Key Laboratory of Crop Genetic Improvement and National Center of Plant Gene Research (Wuhan), Huazhong Agricultural University, Wuhan, 430070 China; 2 Key Laboratory of Biological Resources Protection and Utilization of Hubei Province, Hubei University for Nationalities, Enshi, 44500 China; 3 Collage of Biological Science and Technology, Hubei University for Nationalities, Enshi, 44500 China

## Abstract

Selenium (Se) is an essential mineral element for animals and humans. *Cardamine hupingshanensis* (Brassicaceae), found in the Wuling mountain area of China, has been identified as a novel Se hyperaccumulator plant. However, the mechanism for selenium tolerance in *Cardamine* plants remains unknown. In this study, two cDNA libraries were constructed from seedlings of *C. hupingshanensis* treated with selenite. Approximately 100 million clean sequencing reads were *de novo* assembled into 48,989 unigenes, of which 39,579 and 33,510 were expressed in the roots and leaves, respectively. Biological pathways and candidate genes involved in selenium tolerance mechanisms were identified. Differential expression analysis identified 25 genes located in four pathways that were significantly responsive to selenite in *C. hupingshanensis* seedlings. The results of RNA sequencing (RNA-Seq) and quantitative real-time PCR (RT-qPCR) confirmed that storage function, oxidation, transamination and selenation play very important roles in the selenium tolerance in *C. hupingshanensis*. Furthermore, a different degradation pathway synthesizing malformed or deformed selenoproteins increased selenium tolerance at different selenite concentrations. This study provides novel insights into the mechanisms of selenium tolerance in a hyperaccumulator plant, and should serve as a rich gene resource for *C. hupingshanensis*.

## Introduction

Selenium (Se) is an essential trace element for animals and humans that can be acquired from plant accumulators growing in seleniferous soil. According to tolerance and accumulation quantities of Se, plants can be categorized into three groups: <100 mg Se kg^−1^, 100–1000 mg Se kg^−1^ and 1000–15000 mg Se kg^−1^. Plants which can tolerate or accumulate Se at a concentration of 1000–15000 mg Se kg^−1^ are called Se hyperaccumulators^1^. Most species known to hyperaccumulate Se belong to the Fabaceae family. The ability of hyperaccumulation of Se in plants has evolved several times within the Asteraceae, Brassicaceae and Fabaceae^1^. *Astragalus bisulcatus*, *Stanleya pinnata* and *Symphyotrichum ericoides* are the most widely studied Se hyperaccumulators^2–7^.

*Cardamine hupingshanensis* is a novel hyperaccumulator plant found in the Wuling mountain area. Bai *et al*.^8^ found that *C. hupingshanensis* is primarily distributed in Hunan province in China, at 800–1400 m. However, we found it grows where there is a cloudy slope or valley with coal gangue and running water in the city of Enshi as well as in the counties of Xuan’en, Changyang and Wufeng in Hubei province at 800–1900 m. Yuan *et al*. (2013) and Shao *et al*. (2014) measured concentrations of total Se by hydride generation-atomic fluorescence spectrometry (HG-AFS) and HPLC-ICP-MS, respectively. These studies showed that *C. hupingshanensis* could accumulate Se in excess of 1400 mg Se kg^−1^ of dry matter in all tissues of seedlings, with most not exceeding 4000 mg Se kg^−1^ of dry matter in roots^9,10^.

**Figure 1:**
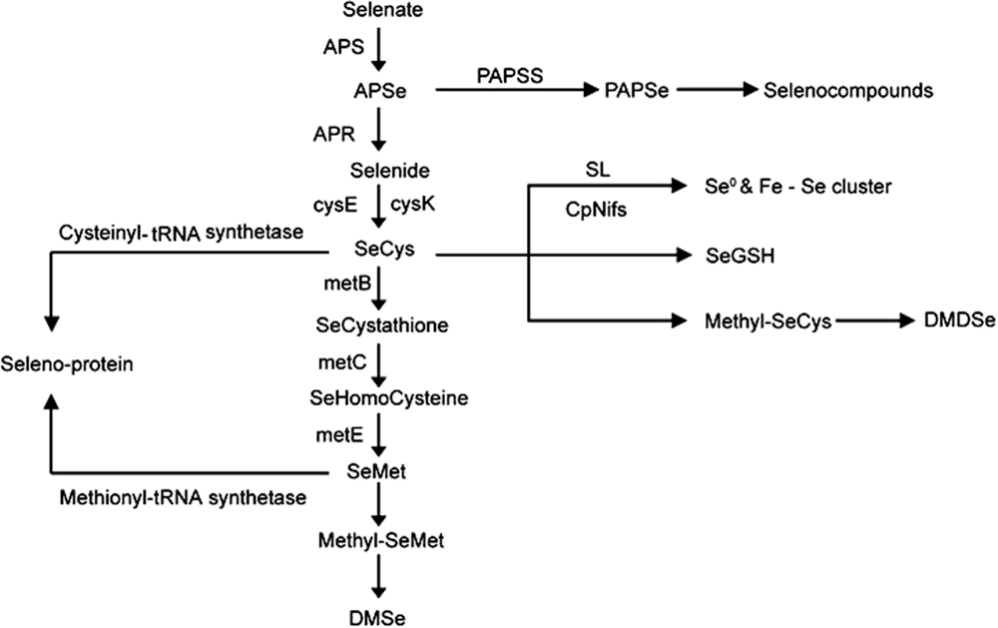
Schematic overview of Se metabolism in plants. Enzyme and metabolites names are abbreviated as follows: APS, Adenosine-5′-phosphsulfate synthetase; APR, adenylylsulfate reductase; PAPSS, 3′-phosphoadenosine 5′-phosphosulfate synthase; cysE, serine O-acetyltransferase; cysK, cysteine synthase A; metB, cystathionine gamma-synthase; metC, cystathionine beta-lyase; metE, 5-methyltetrahydropteroyl- triglutamate-homocysteine methyltransferase; SL, selenocysteine lyase; CpNifS, chloroplastic NifS-like cysteine desulfurase; APSe, Adenosine-5′-phosphoselenate; PAPSe, 3′-Phosphoadenosine 5′-phosphoselenate; SeCys, L-Selenocysteine; SeMet, L-Selenomethionine; Methyl-SeMet,Se-Methylselenomethionine; DMSe, Dimethyl selenide; DMDSe, dimethyl diselenide; SeGSH,selenium-dependent glutathione.

Selenium can be taken up, transported and metabolized by sulfur (S) assimilation pathways because Se is chemically similar to S in plants^1,11,12^. Therefore, inorganic selenium can be assimilated into selenocysteine and selenomethionine, and incorporated into proteins. Several studies have showed that the misincorporation of selenocysteine seems to be the main reason for selenium toxicity^4,11,13,14^. The mechanisms of Se hyperaccumulation and tolerance are based on the common sulfur metabolic pathway but are further focused on special metabolic processes, such as methylation, in *Astragalus bisulcatus* and *Stanleya pinnata*^1–3,15^. The details are described in Fig. 1. The first step is that selenate actived by adenosine-5′-phosphoselenate synthetase (APS), and then the actived selenate on adenosine-5′-phosphoselenate (APSe) reduced by APSe reductase (APR) and converted to selenied. The next step is synthesis of L-Selenocysteine (SeCys) in which serine O-acetyltransferase (cysE) and cysteine synthase A (cysK) catalyzed the integration of selenide. Additionally, 3′-phosphoadenosine 5′-phosphosulfate synthase (PAPSS) leads a branch which formed selenate donor, 3′-phosphoadenosine 5′-phosphoselenate (PAPSe), it can be used to selenation for biomolecules. When selenate and selenite are reduced to SeCys, there are three steps that will result in non-specific incorporation of Se into proteins: first, the selenocysteine lyase (SL) and NifS-like enzymes (CpNifS) specifically break SeCys; second, SeCys methylation mediated by methyltrans- ferases (SMTs) is another important approach for seleniun detoxification and can increase the concentration of internal Se; third, cystathionine-γ-synthase (CγS) converts methylated SeCys to volatile DMSe^16^. The other researchers also detected appreciable concentrations of seleno-glucosinolates and their Se aglycons and selenosugars, possible chemical components of cell wall origin, in selenized plants^1,17–21^.

Despite of the recognized superiority of selenium tolerance in *C. hupingshanensis*, the mechanisms underlying selenium tolerance remain unclear. Here, we performed a *de novo* transcriptome assembly in *C. hupingshanensis* and conducted a comparative transcriptome analysis to explore the putative mechanisms. Our study should be a useful reference for studying selenium tolerance in plants.

## Results

### Transcriptome characteristics in *C*. *hupingshanensis*

**Figure 2:**
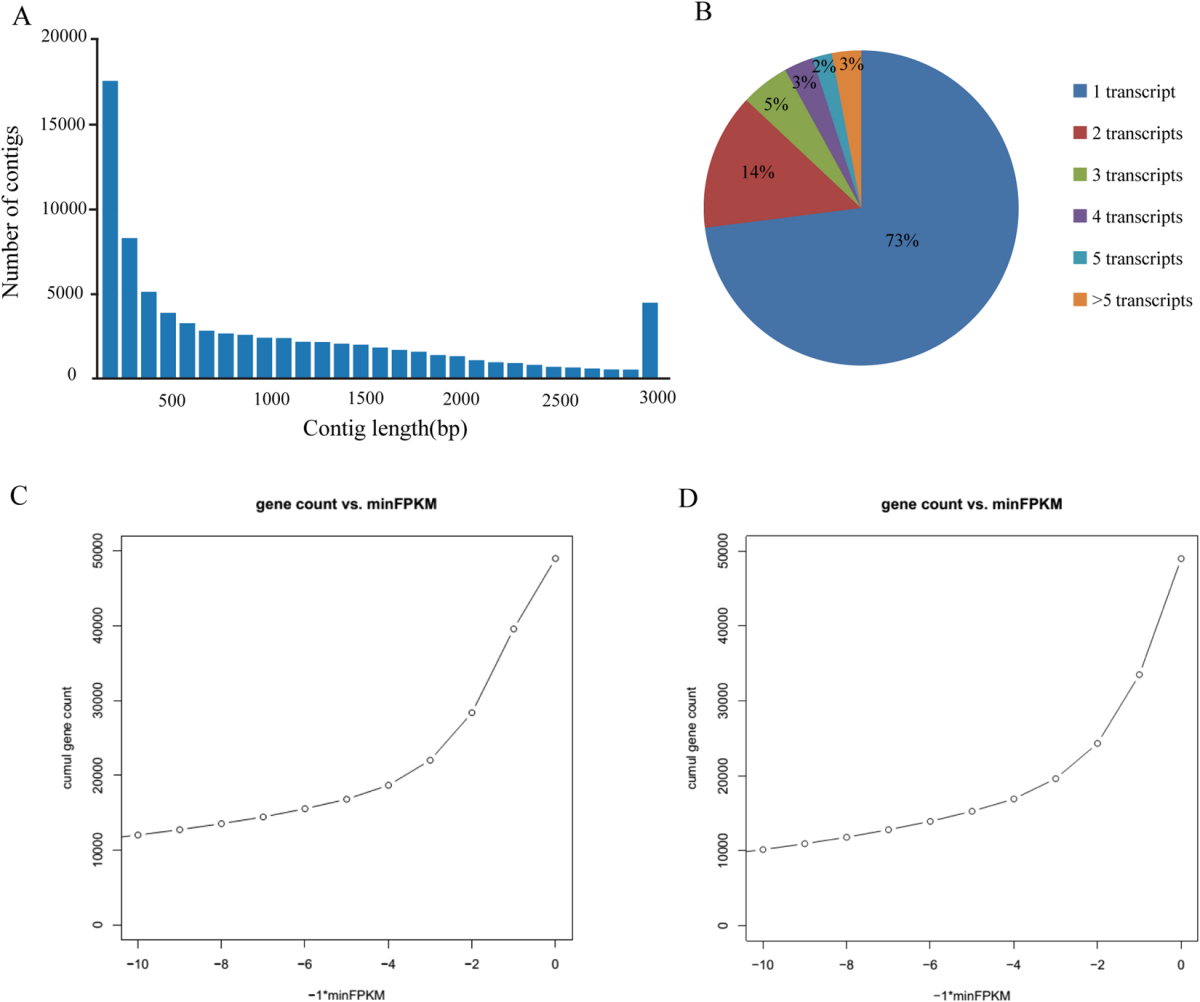
Transcriptome characteristics in *C. hupingshanensis*. The length distribution (**A**) and copies (**B**) distribution of transcripts of *C. Hupingshanensis*. The abundance of transcripts in roots (**C**) and leaves (**D**) of *C. Hupingshanensis*.

**Figure 3:**
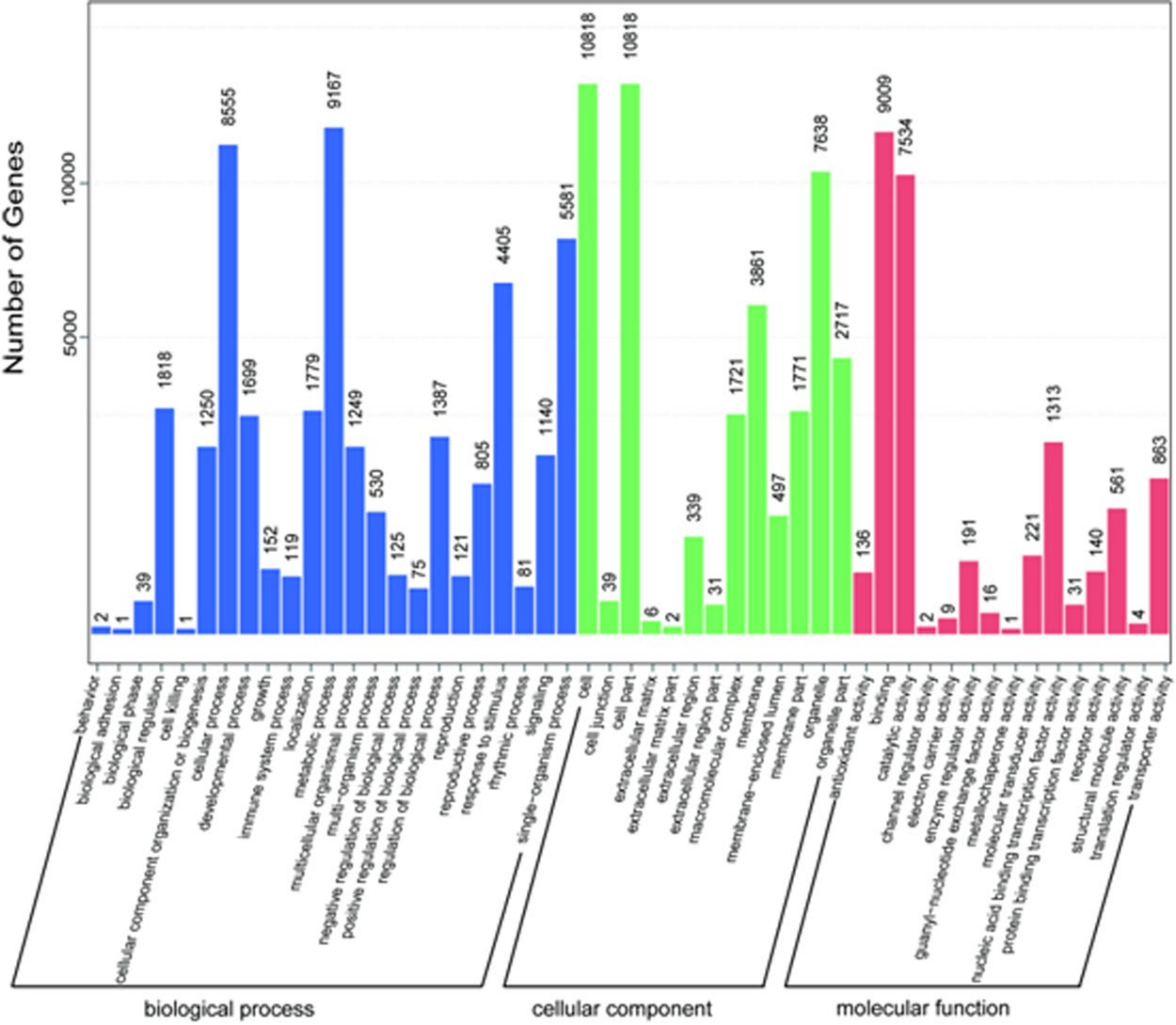
GO functional classification of *C. hupingshanensis* unigenes. Gene Ontology (GO) terms are summarized in three main categories of biological process, molecular function and cellular component.

RNA samples from leaves and roots of *C. hupingshanensis* were prepared for library construction and subsequently sequenced on the Illumina HiSeq. 2500 platform. We obtained a total of 54,765,658 and 50,352,860 raw paired-end reads in leaves and roots, respectively. All sequencing data were deposited in the NCBI database and can be accessed with the Sequence Read Archive (SRA) number of SRP097726. After quality analysis and data filtering, 52,019,342 and 48,078,676 clean reads were retained with Q20 values of 99.0% and 98.9% and GC contents of 45.3% and 47.3% in the leaves and roots, respectively. We performed a *de novo* transcript assembly using these paired-end data to obtain transcript sequences. A total of 78,471 transcripts (average GC content of 41.18%), including 48,989 unigenes, were assembled with a total length of 86,620,844 bp (average transcript length of 1,104 bp). The size and copy distribution of the transcripts are displayed in Fig. 2a and b. The transcript abundance analyzed by bowtie (version 2.23) and RSEM (version 1.2.15) showed that 39,579 and 33,510 transcripts were expressed in the roots and leaves, respectively (displayed in Fig. 2c and d). The function of each unigene set in *C. hupingshanensis* was then annotated by Trinotate (version r20131110) based on homologies to putative or known sequences available in public databases (Table 1). In addition, a gene ontology (GO) analysis, which is a major bioinformatic approach utilizing to represent properties of gene and gene products across all species, was then carried out on the putative proteins. All unigenes were annotated using three ontologies, including biological process (BP), molecular function (MF) and cell component (CC) (Fig. 3). There was a total of 51 different sublevels narrowed down to form the three ontologies. According to the explanations in the non-redundant protein (NR) and the Pfam databases, 48,989 unigenes properly fit into one or more ontologies.

**Table 1:** Annotation results of *C. hupingshanensis* unigenes according to different databases.

Item	Counts	Percentage
All_transcripts	78,471	100.0%
Annotated_transcripts	58,470	74.5%
Top_BLASTX_hit	43,240	55.1%
Top_BLASTP_hit	38,386	48.9%
Pfam	37,041	47.2%
RNAMMER	7	0.0%
SignalP	3,650	4.7%
TmHMM	11,624	14.8%
eggnog	22,074	28.1%
gene_ontology	36,936	47.1%

**Figure 4:**
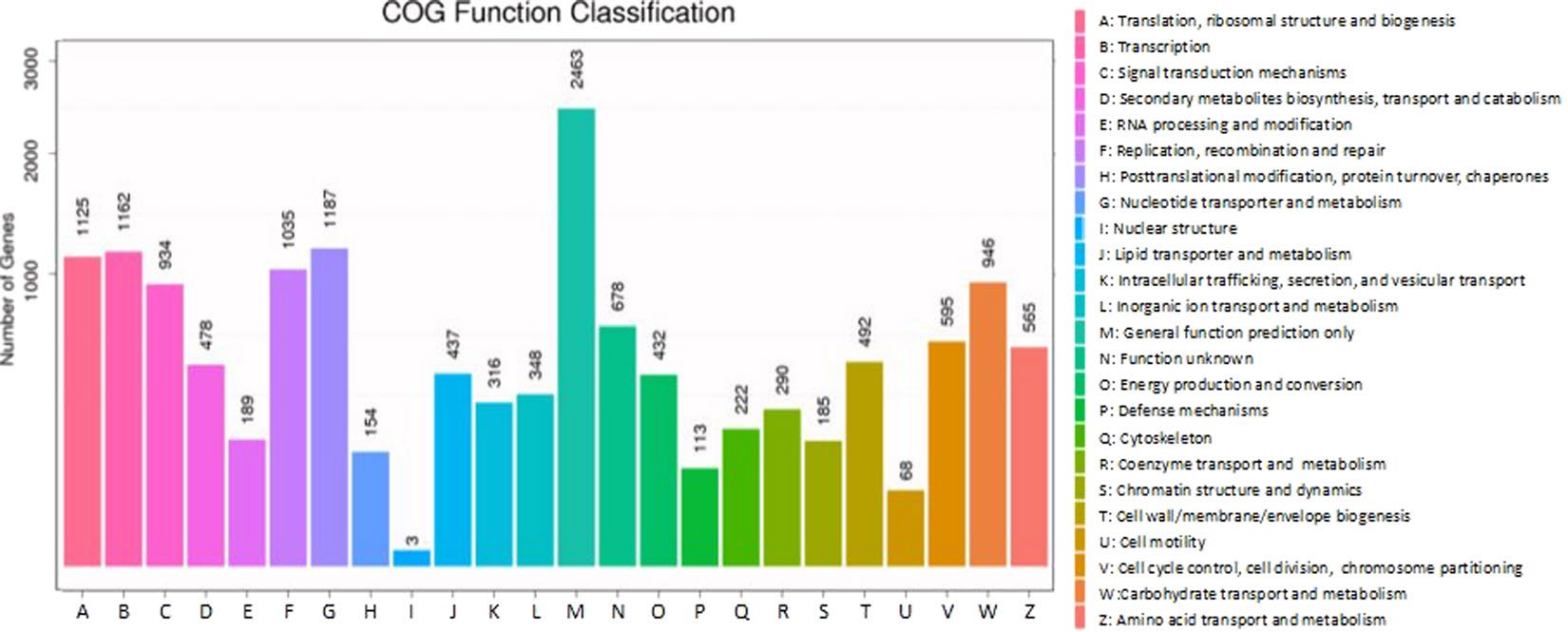
Functional classification of *C. hupingshanensis* unigenes according to COG database.

There were 23 BP subcategories with 40,081 unigenes, 13 CC subcategories with 36,783 unigenes and 15 MF subcategories with 20,031 unigenes. Consistent with findings in other plants, the metabolic process, cellular process and single cell process ontologies in BP were the top three gene ontology terms, with 9,167, 8,555 and 5,581 unigenes, respectively^22,23^. Cell, cell part and organelle terms in CC were the top three classes with 10,818, 10,818 and 7,638 unigenes, respectively. Catalytic activity, nucleic acid binding and transcription factor activity in MF were the top three GO terms with 9,009, 7,543 and 1,313 unigenes, respectively. The COG function classification of the *C. hupingshanensis* unigenes is displayed in Fig. 4. Overall, 14,417 of 48,989 unigenes matched to the COG database were clustered into 24 functional clusters. According to the number of genes, the most significant cluster was the general function prediction only cluster (2,463, 17.08%), followed by the nucleotide transporter and metabolism (1,187, 8.23%); transcription (1,162, 8.06%); translation, ribosomal structure and biogenesis (1,125, 7.80%); and replication, recombination and repair clusters (1,035, 7.18%).

**Figure 5:**
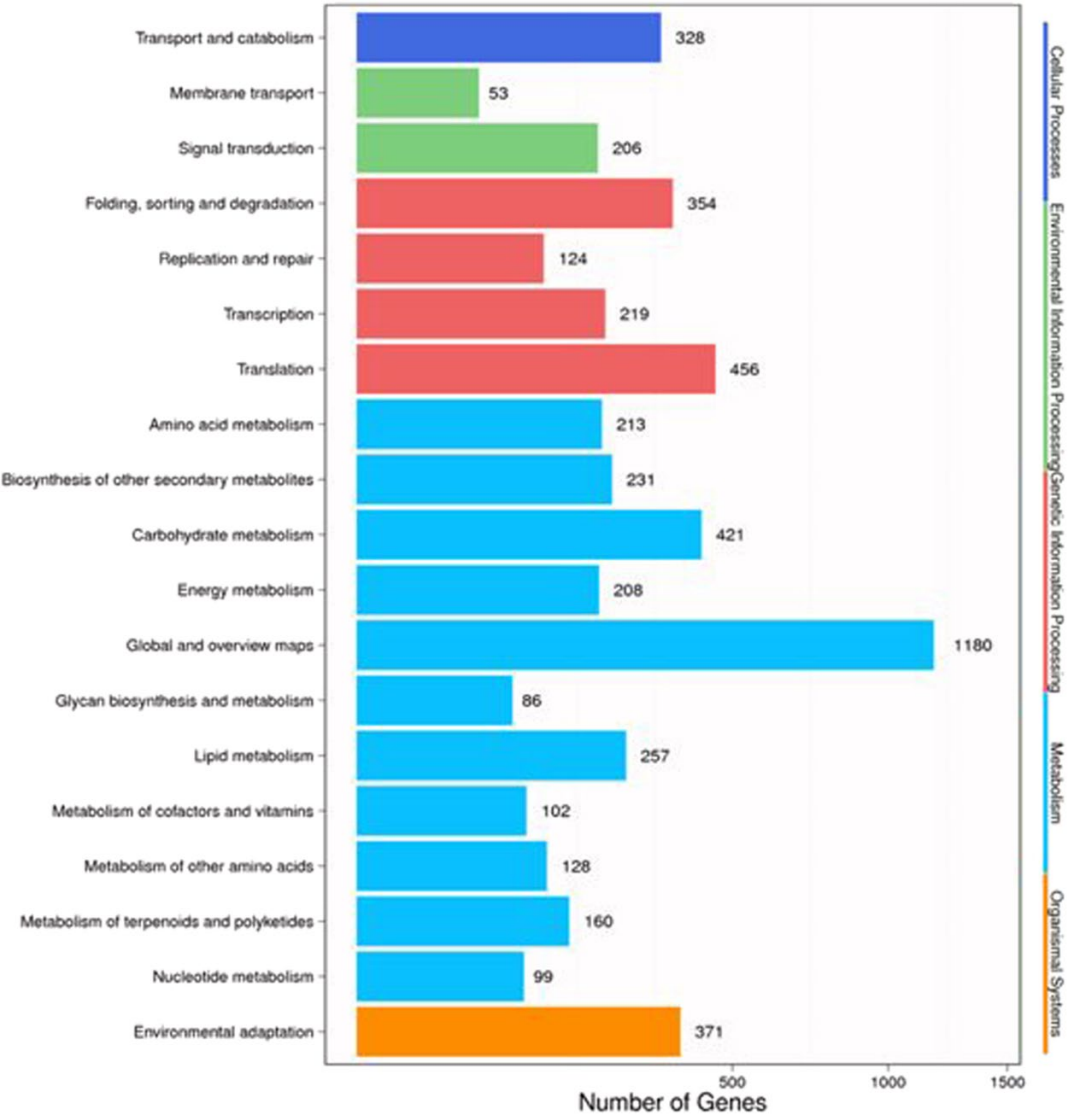
KEGG pathway mapping for *C. hupingshanensis*.

To understand the functions and products of unigenes in putative metabolic pathways, the Kyoto Encyclopedia of Genes and Genomes (KEGG) was used to systematically analyze all unigenes in *C. hupingshanensis* (Fig. 5). A total of 5,196 unigenes obtained in this study were classified into five branches: cellular processes, environmental information processing, genetic information processing, metabolism, and organismal systems. More than half of those aligned with KEGG transcripts were classified into metabolism (59.37%), and 22.19% were classified into environmental information processing. The other highly represented pathways included the global and overview map (1,180, 22.71%), translation (546, 8.78%), carbohydrate metabolism (421, 8.10%), environmental adaptation (371, 7.14%), and folding, sorting, and degradation (354, 6.81%) pathways.

### General DEGs at all selenium concentrations

**Figure 6:**
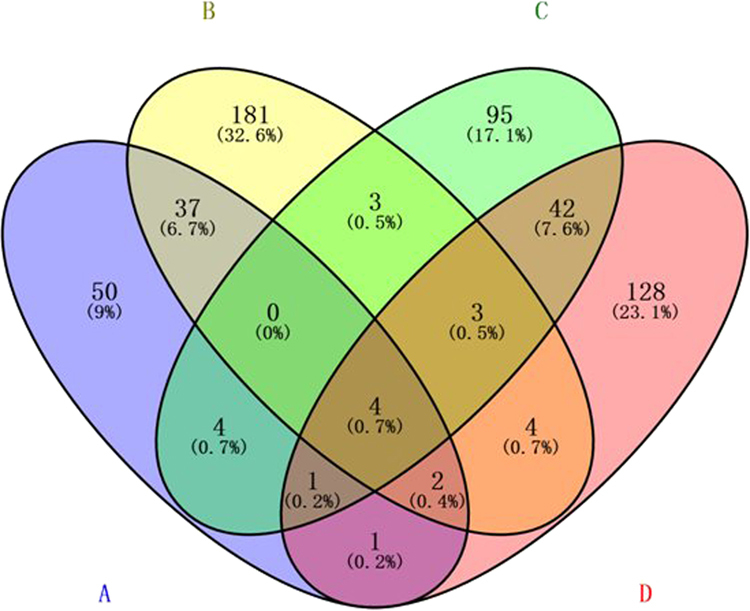
The significantly DEGs responsive to both two Se treated concentration in seedling of *C. hupingshanensis*. The significantly DEGs in roots of seedling between 100 μg Se/L (**A**) and 80,000 μg Se/L (**B**) treated concentration and control. The significantly DEGs in leaves of seedlingbetween 100 μg Se/L (**C**) and 80,000 μg Se/L (**D**) treated concentration and control.

To understand the mechanisms of selenium tolerance in the hyperaccumulator plant *C. hupingshanensis*, another eighteen libraries (one sample including 3 biological replicates, nine libraries for roots and nine libraries for leaves) of seedlings were constructed to identify differentially expressed genes (DEGs) between the control and low Se treatment (100 μg Se/L, slightly higher than the concentration of Se in the water of the high-Se area, treated for 24 hours) and between the control and high Se treatment (80,000 μg Se/L, a stress concentration, treated 24 hours). Using a probability value of more than 0.8 and a minimal FPKM value of 3 (FPKM values used to define up- and down-regulated genes between treatment and control), the overlapping parts and exclusive sections of 670 unigenes from the four comparative groups are shown in Fig. 6. There were 50, 181, 95, and 128 unigenes annotated exclusively in these four groups. There were 4 unigenes that aggregated into a collection from the four groups (Supplementary Table S1). There were 43 and 50 unigenes transcribed in both the roots and leaves at two selenium concentrations, respectively. The roots and leaves shared 9 and 13 unigenes at low and high selenium concentrations, respectively.

Using a probability value of more than 0.8 and a minimal FPKM value of 10 (FPKM values used to define up- and down-regulated genes between treatment and control), 31 and 64 genes were associated with Se response in the root tissue, and 30 (23 genes up-regulated and 7 genes down-regulated) and 103 (42 genes up and 61 genes down) genes were associated with Se response in leaf tissue of seedlings treated with low and high concentrations of Se, respectively. At both concentrations of Se treatment, 6 annotated genes were up-regulated and 1 was down-regulated in root tissue, and 8 genes (including 3 un-annotated genes) were up-regulated and 6 (including 1 un-annotated gene) were down-regulated in the leaf tissue (Table 2).

**Table 2:** The DEGs responsive to both two Se treated concentration. L: low Se treatment concentration, H: high Se treatment concentration.

No.	GeneID	log2Ratio (L/H)	up/down	Tissue	KEGG/Nr/Swiss-Prot annotation
1	c25587_g1_i1	3.67/3.37	up	Root	sulfite oxidase
2	c8451_g1_i1	4.61/3.26	up	Root	LOB domain-containing protein 16
3	c23603_g1_i2	4.02/71.02	up	Root	aspartate aminotransferase, chloroplastic
4	c4063_g1_i1	4.76/5.02	up	Root	thioesterase family protein [Arabidopsis thaliana]
5	c23549_g1_i2	7.77/6.10	up	Root	pyridoxine 4-dehydrogenase
6	c22557_g2_i2	3.64/3.39	up	Root	ATP-binding cassette, subfamily B (MDR/TAP), member 1
7	c16776_g1_i1	2.17/2.25	up	Leave	glutathione S-transferase 12
8	c15496_g1_i1	3.66/3.83	up	Leave	ATGOLS3
9	c24366_g2_i1	4.94/7.17	up	Leave	aryl sulfotransferase
10	c19266_g1_i9	6.30/7.70	up	Leave	cold-inducible RNA-binding protein
11	c26649_g1_i3	5.26/5.19	up	Leave	ATP-binding cassette, subfamily C (CFTR/MRP), member 2
12	c23568_g2_i1	4.14/4.46	up	Leave	regulation of gene expression
13	c22186_g1_i5	11.68/13.03	up	Leave	Dormancy/auxin associated protein
14	c24788_g1_i2	5.23/4.70	up	Leave	none
15	c15033_g1_i1	2.11/2.321	up	Leave	glutathione S-transferase tau 4 (GST-u4)
16	c36512_g1_i1	−7.57/−10.30	down	Root	defensin-like protein 205 [Arabidopsis thaliana]
17	c33674_g1_i1	−3.31/−4.63	down	Leave	none
18	c9285_g2_i1	−4.07/−5.72	down	Leave	none
19	c19001_g1_i2	−3.57/−3.70	down	Leave	xyloglucan: xyloglucosyl transferase
20	c10770_g1_i1	−10.81/−10.81	down	Leave	5′-AMP-activated protein kinase, regulatory beta subunit
21	c15335_g1_i2	−10.57/−10.57	down	Leave	none
22	c26522_g1_i1	−10.37/−10.36	down	Leave	tubulin beta
23	c22186_g1_i6	−10.17/−10.17	down	Leave	expressed protein
24	c10770_g2_i1	−9.99/−9.99	down	Leave	2,4-dihydroxy-1,4-benzoxazin-3-one-glucoside dioxygenase

**Figure 7:**
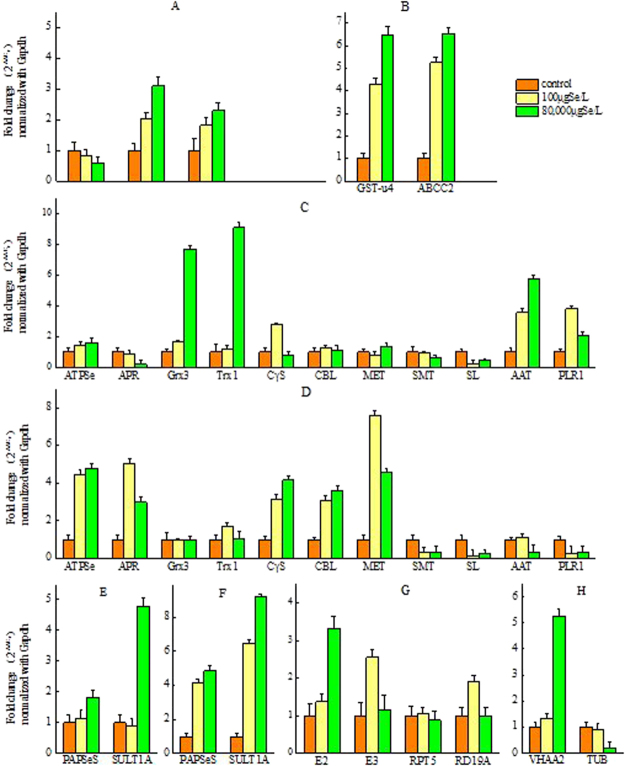
RT-qPCR analysis of the genes related to selenium tolerance mechan- isms in hyperaccumulator plant *C. hupingshanensis* seedling. (**A**,**C**,**E** and **G**) Descripted changes of the genes in root; (**B**,**D**,**F** and **H**) descripted changes of the genes in leaves.

In this study, the expression of *sulfite oxidase* (*SOX*) gene in the root was up-regulated when selenite was added to the culture solution of the *C. hupingshanensis* seedlings (Table 2 and Fig. 7A), suggesting that selenite may be converted to selenate first and then the selenate continued through metabolism.

Vacuole is of great importance because of its storage function, which was demonstrated by the up-regulation of genes in metabolic and transport pathways. Based on the data from RNA-Seq and RT-qPCR (Table 2, Fig. 7A and B), the changes in genes of the glutathione S-transferase family and the C subfamily of the ATP-binding cassette transporters (ABCC) provided direct evidence. The glutathione S-transferase family gene GST-u4 in leaves, which promotes glutathione-chelated selenate to form phytochelatins (PCs)^24^, was up-regulated. The expression of ABCC2 was significantly up-regulated in leaves of *C. hupingshanensis* seedlings when the seedlings were treated with selenite. At the same time, there were four up-regulated genes associated with metal ion binding.

The results of RNA-Seq and RT-qPCR (Table 3, Fig. 7C) showed that the oxidation and transamination of SeCys might be two important processes for Se detoxification in the roots, but the results of RT-qPCR (Fig. 7D) suggested that conversion of SeCys to SeMet was also an important process for Se detoxification in the leaves. The transamination of SeCys or its oxides, such as L-cysteate and L-cysteine-sulfinate, has not been studied extensively in plants. The aspartate aminotransferase (chloroplastic, Aat), a pyridoxal phosphate dependent amino acid aminotransferase, which also located in cysteine and methionine metabolism pathway, could catalyze SeCys, L-cysteate and L-cystiene-sulfinate deaminize^25–27^. It was found that the expression of Aat and pyridoxine 4-dehydrogenase (PLR1) were up-regulated in the roots. Cystathionine gamma-synthase (CγS), cystathionine beta-lyase (CBL) and 5-methyltetrahydropteroyl-triglutamate-homocysteine methyltransferase (MET), which are three key enzymes in the methionine biosynthesis pathway, were found to be up-regulated by RT-qPCR at all concentrations of selenium treatment in leaves (Fig. 7D).

**Table 3:** The significantly change transcripts treated by low selenium concentration in *C. hupingshanensis* seedling.

No.	Gene ID	log2 Ratio	Probability	up/down	Tissue	KEGG/Nr/Swiss-Prot annotation
1	c44959_g1_i1	3.35	0.801	up	Root	VQ motif-containing protein
2	c9362_g1_i1	4.87	0.807	up	Root	E3 ubiquitin-protein ligase RNF13
3	c4774_g1_i1	5.55	0.830	up	Root	E3 ubiquitin-protein ligase MUL1
4	c41336_g1_i1	10.48	0.952	up	Root	E3 ubiquitin-protein ligase MUL1
5	c19839_g2_i3	9.98	0.931	up	Root	Cysteine protease RD19A
6	c8804_g1_i1	5.01	0.828	up	Root	solute carrier family 25, member 44
7	c12744_g2_i1	4.63	0.820	up	Root	expressed protein
8	c24350_g2_i4	4.54	0.814	up	Root	phosphoenolpyruvate carboxykinase (ATP)
9	c21128_g2_i3	9.50	0.904	up	Root	putative S locus-linked protein
10	c22829_g1_i1	3.56	0.805	up	Leave	beta-amylase
11	c17901_g1_i1	3.47	0.803	up	Leave	beta-glucosidase
12	c26996_g1_i2	3.62	0.801	up	Leave	GIGANTEA
13	c25286_g1_i2	3.68	0.801	up	Leave	pseudo-response regulator 5
14	c25286_g1_i1	4.70	0.802	up	Leave	pseudo-response regulator 5
15	c20629_g1_i2	10.33	0.958	up	Leave	two-component response regulator ARR-B family
16	c17041_g1_i3	10.10	0.948	up	Leave	saposin
17	c25623_g1_i2	7.54	0.917	up	Leave	ATP-dependent RNA helicase/ATP-dependent RNA helicase DDX5/DBP2
18	c22072_g1_i3	−4.98	0.811	down	Root	ribulose-bisphosphate carboxylase small chain
19	c18750_g1_i2	−10.20	0.942	down	Root	brassinosteroid insensitive 1-associated receptor kinase 1
20	c26559_g1_i3	−10.16	0.940	down	Root	time for coffee
21	c22072_g1_i2	−10.09	0.937	down	Root	ribulose-bisphosphate carboxylase small chain
22	c19954_g1_i4	−9.92	0.929	down	Root	endonuclease 2
23	c10770_g1_i1	−6.35	0.847	down	Root	2,4-dihydroxy-1,4-benzoxazin-3-one-glucoside dioxygenase
24	c22144_g1_i3	−5.96	0.874	down	Leave	golgin subfamily A member 6-like protein 22
25	c18949_g1_i3	−9.97	0.943	down	Leave	EUTSA_v10006302mg

Selenation which is similar to sulfation in plants, is another important pathway for detoxification of selenium because it can reduce SeCys biosynthesis. The aryl sulfotransferase enzyme (SULT1A, EC 2.8.2.1) is a member of the sulfotransferase (SOT, EC 2.8.2.-) protein family. This enzyme can transfer a sulfate group from the donor 3′-phosphoadenosine 5′-phosphosulfate (PAPS) to phenolic sulfate esters to a phenolic acceptor substrate in the sulfur and glucosinolate metabolism pathway. According to the KEGG analysis, this enzyme plays a key role in the selenation pathway^28^. However, aryl sulfotransferase is present in animals and prokaryotes, while aryl sulfotransferase is found only in *R. communis* L.^28–30^. The expression of SULT1A increased 4.94 (100 μg Se/L)- and 7.17 (80,000 μg Se/L)-fold in the leaves (Table 2 and Fig. 7F). That is, the response of selenation is a common pathway in leaves of *C. hupingshanensis* when seedlings are treated with selenium. Therefore, we can conclude that selenation is the more common approach of tolerating selenium stress in leaves of *C. hupingshanensis* seedlings.

The other genes that significantly responded to Se are involved in multiple physiological processes. The gene for transcription factor LBD 16 of the LBD family, which plays crucial roles in diverse growth and development processes, including the establishment and maintenance of the developmental boundary of lateral organs^31^, was up-regulated in the roots, indicating that selenium could promote lateral root formation in plants. AtGoLS3 is a member of the galactinol synthase (GolS) family, which initiates the biosynthesis of raffinose oligosaccharides (RFO), may act as an osmoprotectant in drought stress tolerance through UDP-galactose^32,33^. According to results of RNA-Seq, a novel possibility is that AtGoLS3 is induced not only by cold stress but also by selenium stress. The cellular-localized cold-inducible RNA-binding protein, also called the glycine-rich RNA-binding protein, which appears to be involved in the adaptation of abiotic and biotic stress^34^, was another significantly up-regulated gene that strongly responded to selenium in the leaves. Another significantly up-regulated gene that responded to selenium was that coding for the dormancy/auxin-associated protein, which is involved in growth suppression in bud and hypocotyl tissues and whose expression increases in response to abiotic or biotic factors^35^. However, xyloglucan xyloglucosyl transferase, also called xyloglucan endo-transglycosylase (XET), which is involved in wall-loosening, wall-strengthening process, gravitropic responses and the incorporation of nascent xyloglucan into the wall during biosynthesis^36^, was significantly down-regulated at all concentrations of selenium. The down-regulated regulatory beta subunit was one of three subunits of 5′-AMP-activated protein kinase (AMPK), which plays a role in maintaining cellular energy homeostasis^37^. Selenium also affected the process of phagolysosome formation by down-regulation of tubulin beta units of the microtubule.

### Special DEGs at low concentrations of Se treatment

The same criteria described above were used to analyze special DEGs in the seedlings of *C. hupingshanensis* treated with low Se concentrations (100 μg Se/L). There were 9 up-regulated genes and 5 down-regulated genes that were found to be specifically expressed in the roots, and 7 up-regulated genes and 2 down-regulated genes were found to be specifically expressed in the leaves. The low Se concentration treatment not only significantly increased the storage function of the vacuole as well as selenation and transamination but also invoked protein degradation and other physiological responses (Table 3 and Fig. 7G). E3 ubiquitin-protein ligase RNF13 and MUL1, which are two important members of the ubiquitin–proteasome pathway (UPP), were significantly up-regulated in the roots under low Se treatment. RING finger protein 13 (RNF13) is a member of the largest family of ubiquitin ligases in eukaryotes and is an ER/Golgi membrane-associated E3 ubiquitin ligase that has been identified as a novel RING-based ubiquitin ligase^38,39^. Mitochondrial E3 ubiquitin protein ligase 1 (MUL1), which is localized to the mitochondria, is a crucial moderator of retinoic acid-inducible-gene I (RIG-I) signaling^40^. Cysteine protease RD19A, which is an important enzyme of proteolysis involved in cellular protein catabolic processes and responses to osmotic and salt stress that is located mainly in the vacuole and lysosome^41^, was also up-regulated (Table 3 and Fig. 7G). The low concentration of Se treatment significantly affected carbohydrate metabolism by up-regulating phosphoenolpyruvate carboxykinase and the small chain of ribulose-bisphosphate carboxylase in the roots and beta-amylase and beta-glucosidase in the leaves. Some genes related to the circadian clock, such as GIGANTEA (GI)^42,43^ and pseudo-response regulator 5 (PRR5)^44^ as well as those of the two-component response regulator ARR-B family (PCL1)^45,46^, were up-regulated, which may suggest effects of selenium on the circadian clock in leaves of *C. hupingshanensis*. The two least up-regulated genes were saposin and ATP-dependent RNA helicase DDX5/DBP2, and DDX5/DBP2 act in the process of nonsense-mediated mRNA decay and ribosome biogenesis through rRNA. A few genes including brassinosteroid insensitive 1-associated receptor kinase 1, time for coffee, endonuclease 2 and ribulose-bisphosphate carboxylase small chain were inhibited by low Se treatment in the roots. Only golgin subfamily A member 6-like protein 22 and a putative transcription factor were down-regulated in the leaves.

### Special DEGs at high concentrations of Se treatment

The same criteria described above were used to analyze special DEGs in *C. hupingshanensis* seedlings treated by a high concentration of Se (80,000 μg Se/L). Compared with the low Se concentration treatment, there were more genes with expression changes of various physiological processes and cellular functions: 23 genes were up-regulated and 31 genes were down-regulated specifically in the roots, and 22 genes were up-regulated and 12 genes were down-regulated specifically in the leaves (Table 4).

**Table 4:** The significantly change transcripts treated by high selenium concentration in *C. hupingshanensis* seedling.

No.	Gene ID	log2 Ratio	Probability	up/down	Tissue	KEGG/Nr/Swiss-Prot annotation
1	c19857_g1_i1	2.82	0.801	up	Root	ubiquitin-conjugating enzyme E2 7
2	c24366_g2_i1	4.05	0.819	up	Root	aryl sulfotransferase
3	c14018_g1_i1	3.81	0.816	up	Root	defensin
4	c19092_g1_i3	3.22	0.808	up	Root	tetratricopeptide repeat domain-containing protein
5	c16436_g1_i1	3.21	0.808	up	Root	thioredoxin 1
6	c33369_g1_i1	5.11	0.802	up	Root	glutaredoxin C-10
7	c22655_g1_i7	2.93	0.801	up	Root	calmodulin-binding protein
8	c16335_g1_i2	3.31	0.807	up	Root	histone H2A
9	c28060_g1_i1	4.40	0.822	up	Root	protein SPT2
10	c23323_g1_i1	3.20	0.803	up	Root	phosphoribosylamine–glycine ligase
11	c20557_g2_i1	3.55	0.806	up	Root	BREVIPEDICELLUS
12	c20557_g1_i1	4.79	0.825	up	Root	BREVIPEDICELLUS
13	c20557_g1_i3	4.02	0.800	up	Root	BREVIPEDICELLUS
14	c25718_g1_i2	3.89	0.801	up	Root	CCR4-NOT transcription complex subunit 6
15	c44228_g1_i1	5.09	0.824	up	Root	zinc finger protein-like
16	c24721_g3_i5	4.07	0.810	up	Root	phytochrome-interacting factor 3
17	c21356_g1_i1	3.39	0.803	up	Root	auxin-responsive protein IAA
18	c34034_g1_i1	4.48	0.814	up	Root	gibberellin 2-oxidase
19	c13127_g1_i1	4.38	0.817	up	Root	peroxidase 43
20	c8022_g1_i1	3.85	0.804	up	Root	peroxidase 67
21	c18293_g1_i3	4.51	0.820	up	Root	cathepsin A (carboxypeptidase C)
22	c31295_g1_i1	5.80	0.871	up	Root	glycosyl hydrolase family 9 protein
23	c22386_g2_i1	5.32	0.830	up	Root	3,4-dihydroxy 2-butanone 4-phosphate synthase/GTP cyclohydrolase II
24	c24121_g1_i5	10.32	0.954	up	Root	Cd^2+^/Zn^2+^-exporting ATPase
25	c13966_g1_i1	5.33	0.803	up	Root	esterase/lipase/thioesterase family protein
26	c16776_g1_i1	2.17	0.801	up	Root	glutathione S-transferase 12
27	c26122_g2_i1	3.94	0.811	up	Leave	ferritin heavy chain
28	c22426_g1_i1	3.81	0.806	up	Leave	ARALYDRAFT_483040
29	c23861_g1_i3	3.47	0.802	up	Leave	RAV-like factor
30	c15569_g2_i1	3.89	0.806	up	Leave	ATHB-12
31	c15569_g1_i2	5.90	0.846	up	Leave	ATHB-12
32	c24052_g1_i1	4.95	0.821	up	Leave	transcription factor TGA; vacuolar protein 8
33	c21583_g1_i2	5.92	0.843	up	Leave	ANAC019
34	c21583_g1_i9	5.60	0.864	up	Leave	ANAC019
35	c13823_g1_i1	4.30	0.811	up	Leave	NAC transcription factor RD26
36	c21583_g1_i4	4.42	0.801	up	Leave	NAC domain-containing protein 19
37	c10147_g1_i1	5.11	0.838	up	Leave	group I late embryogenesis abundant protein
38	c24183_g1_i2	3.68	0.801	up	Leave	CTP synthase (glutamine metabolic process)
39	c14600_g1_i2	4.41	0.805	up	Leave	CARUB_v10020817mg
40	c23954_g1_i6	5.17	0.826	up	Leave	12-oxophytodienoic acid reductase 1
41	c21478_g1_i2	5.16	0.816	up	Leave	desulfoglucosinolate sulfotransferase A/B/C
42	c5110_g1_i1	6.12	0.852	up	Leave	calcium-binding protein CML
43	c14080_g1_i2	4.26	0.811	up	Leave	ARALYDRAFT_908317
44	c14080_g2_i2	5.62	0.847	up	Leave	EUTSA_v10010032mg
45	c17912_g1_i1	4.94	0.804	up	Leave	CARUB_v10010130mg
46	c14156_g1_i1	5.50	0.826	up	Leave	F21J9.24
47	c25018_g1_i1	5.36	0.847	up	Leave	BnaC08g17590D
48	c22144_g1_i6	10.23	0.949	up	Leave	golgin subfamily A member 6-like protein 22
49	c25889_g1_i1	10.24	0.949	up	Leave	V-type H^+^-transporting ATPase subunit I
50	c18601_g1_i3	4.70	0.812	up	Leave	syntaxin 7
51	c25633_g1_i5	5.48	0.812	up	Leave	protein transport protein SEC. 23
52	c1958_g1_i1	−3.05	0.806	down	Root	pathogenesis-related protein 1
53	c40248_g1_i1	−3.64	0.814	down	Root	cysteine-rich secretory proteins
54	c16712_g3_i1	−2.81	0.800	down	Root	aquaporin PIP
55	c19607_g2_i1	−3.28	0.809	down	Root	ferulate-5-hydroxylase
56	c22389_g1_i2	−2.95	0.801	down	Root	coniferyl-aldehyde dehydrogenase
57	c13360_g1_i2	−3.97	0.818	down	Root	extensin-2-like
58	c20331_g1_i1	−3.46	0.811	down	Root	—
59	c13360_g1_i3	−3.44	0.809	down	Root	—
60	c20331_g3_i1	−3.64	0.810	down	Root	—
61	c21893_g1_i1	−4.23	0.821	down	Root	cytochrome P450 71A12
62	c19961_g1_i1	−2.99	0.802	down	Root	Peroxidase 3
63	c36847_g1_i1	−3.73	0.807	down	Root	Peroxidase 56
64	c6356_g1_i1	−6.08	0.885	down	Root	Peroxidase 11a (BnaAnng21310D)
65	c24169_g1_i3	−3.18	0.805	down	Root	adenylyl-sulfate reductase (glutathione), APR1
66	c13529_g1_i1	−7.08	0.902	down	Root	protein RESPONSE TO LOW SULFUR 3
67	c24828_g3_i1	−4.96	0.840	down	Root	sulfate transporter 1.2
68	c24828_g4_i4	−5.31	0.855	down	Root	sulfate transporter 1.2(F28K19.22)
69	c26204_g1_i2	−3.83	0.812	down	Root	putative cation/hydrogen exchanger
70	c7675_g1_i1	−3.44	0.806	down	Root	myb proto-oncogene protein, plant
71	c16368_g1_i3	−10.31	0.953	down	Root	myb proto-oncogene protein, plant
72	c17444_g1_i1	−3.58	0.809	down	Root	chitinase
73	c11828_g2_i1	−3.64	0.807	down	Root	FAD-binding domain-containing protein
74	c9067_g1_i1	−3.78	0.805	down	Root	respiratory burst oxidase-B
75	c18905_g2_i1	−3.67	0.803	down	Root	serine/threonine-protein kinase PBS1
76	c19857_g1_i2	−5.39	0.847	down	Root	ubiquitin-conjugating enzyme E2 G1
77	c44867_g1_i1	−5.57	0.858	down	Root	disease resistance response/ dirigent - like protein
78	c22386_g2_i3	−6.86	0.918	down	Root	3,4-dihydroxy 2-butanone 4-phosphate synthase/GTP cyclohydrolase II
79	c25447_g1_i5	−7.45	0.937	down	Root	cyclic nucleotide gated channel, other eukaryote
80	c18259_g2_i2	−4.97	0.807	down	Root	senescence-associated protein
81	c33681_g1_i1	−5.31	0.823	down	Root	cathepsin L
82	c19954_g1_i4	−9.92	0.938	down	Root	endonuclease 1
83	c27203_g3_i1	−6.31	0.814	down	Root	longifolia 1
84	c9050_g2_i1	−3.39	0.803	down	Leave	Cell wall-associated hydrolase
85	c9050_g3_i1	−3.93	0.811	down	Leave	Mitochondrial protein
86	c10003_g1_i1	−3.38	0.803	down	Leave	Ribosomal protein S10
87	c8377_g2_i1	−5.45	0.863	down	Leave	PSI P700 apoprotein A2
88	c8377_g1_i1	−5.21	0.849	down	Leave	photosystem I P700 apoprotein A1
89	c36534_g1_i1	−3.61	0.805	down	Leave	ribulose-1,5-bisphosphate carboxylase/oxygenase large subunit
90	c36532_g1_i1	−3.64	0.803	down	Leave	Photosystem II CP43 chlorophyll apoprotein
91	c32267_g1_i1	−5.30	0.854	down	Leave	senescence-associated protein
92	c17835_g6_i1	−5.35	0.855	down	Leave	cytochrome P450 like_TBP
93	c22145_g1_i2	−4.92	0.821	down	Leave	apocytochrome b
94	c26528_g1_i2	−10.62	0.962	down	Leave	ARALYDRAFT_355122
95	c26586_g1_i3	−10.08	0.943	down	Leave	glycosyl hydrolase family 38 protein

The plant *C. hupingshanensis*, a novel selenium hyperaccumulator, had distinctive reactions to high concentrations of selenium (Table 4 and Fig. 7D). The first reaction was repression of selenium uptake through down-regulating the expression of sulfate transporter 1.2 (*Sultr1;2*) which was a key protein involved in sulfate and selenate transport and expressed mainly in the root cortex, the root tip and lateral roots^47,48^. The second change was repressed reduction of selenate in roots by down-regulating the expression of adenylyl-sulfate reductase (glutathione, APR1), a critical enzyme catalyzing reduction of adenosine 5′ phoshposulfate (APS) or phoshposelenate (APSe), in which C and N terminal domains had a GRX and TRX like function respectively^49^. The last distinctive change comes from the up-regulated expression of aryl sulfotransferase (*SULT1A*), which is closely connected with sulfation or selenation. These results indicated that the flux of selenate on APS was converted to PAPSe and used for selenation, but was not reduced to selenide or combined into SeCys and selenoprotien in the root when *C. hupingshanensis* was treated with high concentrations of selenium^50^.

The visible effects of redox homeostasis when treated with high concentrations of selenium were through the regulation of thioredoxin (TRX) and glutaredoxin (GRX) which were involved in detoxifying reactive oxygen species (ROS) during stress responses and determination protein thiol/disulfide status, and played key roles in the maintenance of cellular redox homeostasis through the sensing and reducing equivalents to a large number of target proteins, such as reductases, peroxidases, transcription factors, metabolic enzymes of glycolysis, and photosynthesis or through structural modifications of target proteins^51,52^. The genes of thioredoxin 1 (*Trx 1*) and glutaredoxin C-10 (*GrxC10*) from the roots were up-regulated (Table 4 and Fig. 7D) when the seedlings of *C. hupingshanensis* were treated by high concentrations of selenite. Simultaneously, peroxidase 43 and 67 which were the important target proteins of TRX, and GRX were up-regulated in the roots^53^.

Regarding photosynthesis, high selenium concentration suppressed the expression of genes involved in light and dark reaction. CP43, one of the components of the core complex of photosystem II (PSII) which binds chlorophyll and helps catalyze the primary light-induced photochemical processes of PSII^54^, was down-regulated when treated with high concentrations of selenium in the leaves. PsaA and PsaB, which bind P700 and are the primary electron donor of photosystem I (PSI), as well as the electron acceptors A0, A1 and FX^55^, were also down-regulated when treated with high concentrations of selenium in the leaves. So, not only the light harvesting process located in PSII but also the transferring of electron process located in PSI of the light reaction of photosynthesis were suppressed when treated with high concentrations of selenium. On the other hand, Rubisco is the key enzyme complex in dark reaction and catalyzes two reactions: the carboxylation of D-ribulose 1,5-bisphosphate, the primary event in carbon dioxide fixation, as well as the oxidative fragmentation of the pentose substrate, but the L subunit of Rubisco was down-regulated when treated with high concentrations of selenium in the leaves. The carbon fixation in dark reaction was also inhibited by high concentrations of selenium through down-regulating the gene expression of the L subunit of Rubisco. In addition, phytochrome-interacting factor 3 (PIF3)^56–58^, which is a basic helix-loop-helix (bHLH) transcription factor closely related to the switch between skotomorphogenesis and photomorphogenesis, was up-regulated in the roots when the seedlings were exposed to light, but the function of PIF3 decided by its state phosphorylation, is still unclear.

The root growth and development of *C. hupingshanensis* seedlings were affected predominantly by high concentrations of selenium. The first change comes from the genes involved in lignin biosynthesis. Five members of class III perooxidases were changed, which play critical roles in lignin biosynthesis, reduction of hydrogen peroxide, auxin and secondary metabolism^53^. The genes of peroxidase 43 and 67 were up-regulated, and peroxidase 3, 11a and 56 were down-regulated in the roots. At the same time, the genes encoding ferulate-5-hydroxylase and coniferyl-aldehyde dehydrogenase in the phenylpropanoid biosynthesis pathway associated with the production of precursors for lignin biosynthesis were down-regulated^59,60^. Additionally, the down-regulation of respiratory burst oxidase homologs (rbohs) decreases the production of superoxide^61^ in the roots. The second change comes from the genes involved in the process of the development of roots. Gibberellin 2-oxidase (GA2ox), which plays very important roles in plant growth and development and can alter expression of lignin biosynthesis-related genes to reduce biomass accumulation and lignification^62^, was up-regulated; it is also involved in resistance to high-salinity stress^63^. All the above changes may indicate that selenium can affect the rigidity and strength of roots. The up-regulation of transcription factor LBD 16, auxin-responsive protein IAA (AUX/IAA), and glycosyl hydrolase family 9 (Cel3) was associated with lateral root initiation and development^64,65^. The down-regulation of four genes including extensin-2-like and one of chitinases indicated that selenium could affect the growth and development of lateral roots significantly.

Another predominant character is that the degradation of protein occurred in the roots and leaves at the same time but in different tissues and subcellular organelles (Table 4 and Fig. 7G and H). The gene of ubiquitin-conjugating enzyme E2, another key member in the UPP, was also up-regulated in the roots. The gene of V-type H^+^-transporting ATPase subunit I (VHA-a2), which is located on mature phagosomes, was up-regulated in the leaves. There were more genes responding to the stress from selenium, such as drought tolerance- and pathogen resistance-related genes. These included defensin, pathogenesis-related protein 1, aquaporin PIP, ferritin heavy chain, ARALYDRAFT_483040 (defense response), ATHB-12, transcription factor TGA, ANAC019, RD26, group I late embryogenesis abundant protein (LEA), and protein transport protein SEC. 23 as well as four metal binding proteins, of which only pathogenesis-related protein 1 and aquaporin PIP were down-regulated in the roots and the others were all up-regulated either in the roots or leaves. Surprisingly, the drought tolerance-related genes ATHB-12^48,66^, ANAC019^67,68^, RD26^69^ and group I LEA^70^ were significantly up-regulated in the leaves, and aquaporin PIP was down-regulated in the roots under the selenium stress. The process of new protein modification was accelerated by up-regulation of Sec. 23, which initiated the COP II coat complex assembly^71^. The pathogen resistance-related genes showed a puzzling change in expression: pathogenesis-related protein 1 was down-regulated in the roots, and transcription factor TGA, whose members interact with the key components (ankyrin repeat protein and non-expresser of pathogen-related (PR) (NPR1) genes) in the SA defense signaling pathway^72^, was up-regulated in the leaves.

Regarding the senescence-associated physiology, the RAV-like factor, which can inhibit the growth of plant leaf, root and stem^73,74^, was up-regulated, and the senescence-associated protein was down-regulated in the leaves.

The SPT2 chromatin protein which was up-regulated in the roots is an important histone chaperone and can facilitate ribosomal DNA transcription through chromatin remodeling^75^. Together with the up-regulation of histone H2A, these results suggest that selenium could function in the process of gene expression in high concentration of Se in the roots. The up-regulation of CCR4-NOT transcription complex subunit 6 also supported this speculation.

## Discussion

### Selenate is the initial compound of selenium metabolism

**Figure 8:**
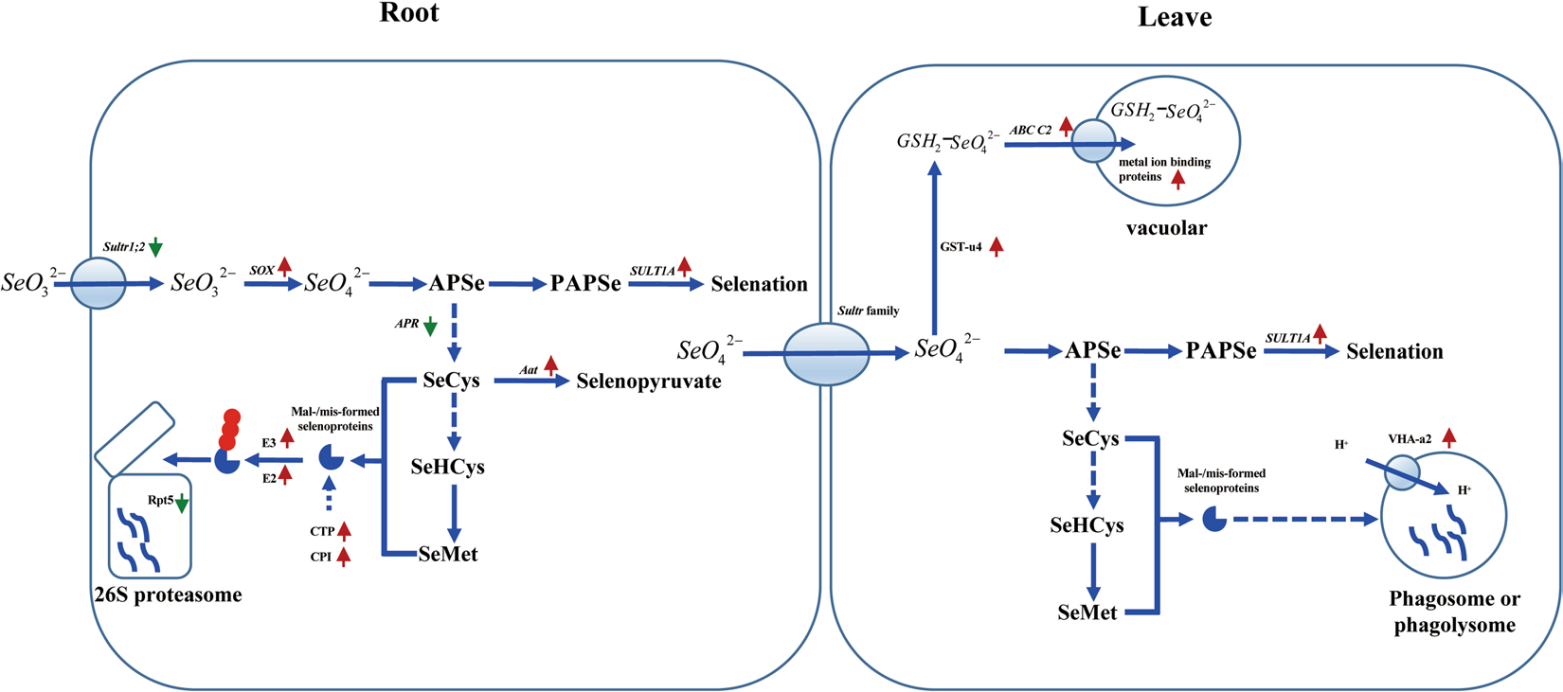
Selenium tolerance mechanisms in hyperaccumulator plant *C. hupingshanensis*.

Selenium is chemically similar to sulfur and is assimilated by plants via the same metabolic pathways^76,77^. Most plants nonspecifically take up selenate from the environment by means of sulfate transporters and assimilate selenate into organic forms of Se via S metabolic pathways^7^. The conversion of selenate to selenite requires the continuous action of two enzymes (Fig. 1). ATP sulfurylase (APS) mediates the binding of selenate with ATP, forming adenosine phosphoselenate (APSe). This compound is then reduced to selenite through APS reductase (APR)^16^. However, we found that SOX was up-regulated when selenite was added to the culture solution of the *C. hupingshanensis* seedlings. Therefore, we can deduce that selenite might be converted to selenate first and then was incorporated into ATP by APS, reduced to selenite by APR, and reduced to selenide before finally being incorporated into SeCys in the root tissue of *C. hupingshanensis* seedlings (Fig. 8).

### The storage function of the vacuole plays an important role in selenium tolerance

**Figure 9:**
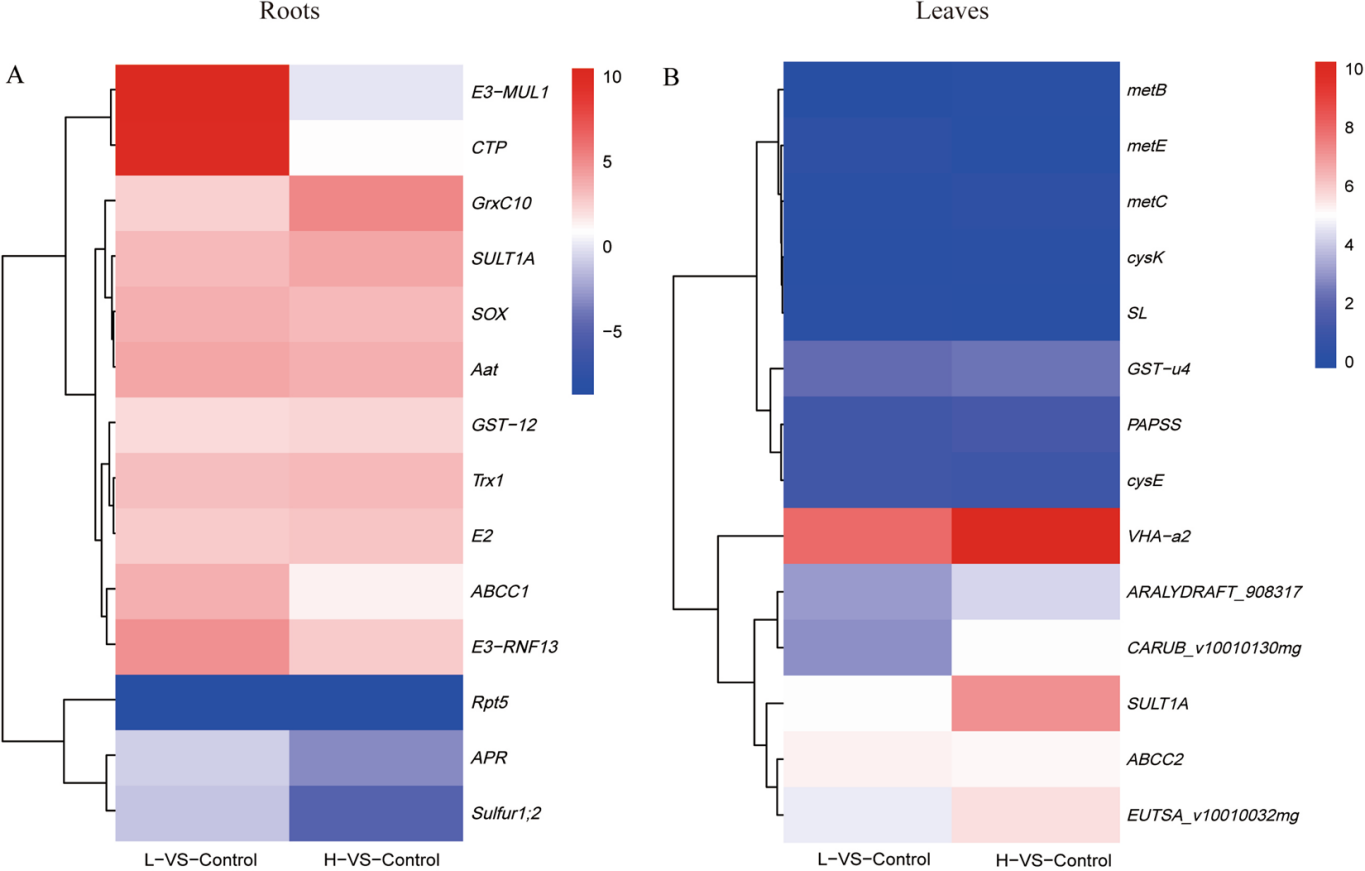
Expression levels of the candidate unigenes coding key enzyme involved in selenium tolerance mechanisms in hyperaccumulator seedling of *C. hupingshanensis*. The candidate unigenes from root (**A**) and leaf (**B**), respectively. Blue and red colors are used to represent low-to-high expression levels, and color scales correspond to the mean centered log2-transformed FPKM values.

After APSe formed, the members of glutathione S-transferase family genes, GST u4 (in leaves) (Figs 8 and 9B,C) were up-regulated to transfer the selenate ion to GSH and to form glutathione-S conjugate (GS-X). ABCC2 (in leaves), belonged to the subfamily C (CFTR/MRP) of ATP-binding cassette superfamily, which proved to be the long-sought and major vacuolar plant PC transporters^78^, were up-regulated. Here, the expression of ABCC2 was significantly up-regulated in the leaves (5.26 folds at 100 μg Se/L and 5.19 folds as 80,000 μg Se/L) of *C. hupingshanensis* seedlings when treated with selenite. Additionally, four genes encoding metal ion binding proteins were up-regulated. Therefore, we deduced that partial selenate was first chelated by glutathione-derived peptides with glutathione sulfur transferase (GST) and was then transported into the vacuole by MRP2 of *C. hupingshanensis* to detoxify and sequester the heavy metal in the roots and leaves. All these results suggest that the storage of the vacuole is an important way to tolerate selenium in *C. hupingshanensis* seedlings.

### Transamination is an important mechanism of selenium detoxification

The fate of SeCys enormously influences the capacity of selenium tolerance in plants. The misincorporation of SeCys was believed to be the main reason for selenium toxicity^4,11,13,14^. Therefore, the fate of SeCys will determine the toxicity. SeCys can be methylated by SeCys methyltransferase (SMT), or it can be converted into SeMet by CγS, oxidized by SL and specifically broken by CpNifS^16^. The process of SeCys conversion to SeMet still plays an important role, as demonstrated by the up-regulation of CγS, CBL and MET in the leaves. However, the transamination of SeCys or its oxides, such as L-cysteate and L-cysteine-sulfinate, has not been studied in plants. In this study, we found that the expression of Aat was up-regulated in the roots compared with the control. Furthermore, PLR1 was also up-regulated in the roots. Therefore, we can deduce that SeCys deamination by Aat is an important pathway for detoxification of selenium in the roots of *C. hupingshanensis* seedlings (Figs 8 and 9A).

### Selenation is the more common mechanism for selenium detoxification

Although it is not the only route, selenation, which is similar to sulfation in plants, is another pathway for detoxification of selenium. APR is a key enzyme in both sulfate and selenate reduction^49^ which was down-regulated with 3.18-fold in the roots. Therefore, the new metabolic pathway to transfer selenate seemed to be more important. Two ways were found for selenate stress: (1) the selenate is chelated by GSH and then transported into the vacuole; (2) the APSe, 3′-phosphoadenosine 5′-phosphoselenate synthase (PAPSeS) and aryl sulfotransferase (SULT1A), which are present in animals and prokaryotes but were found only in *Ricinus communis* L.^28,29^, transfer selenate to a phenolic hydroxy group, forming selenocompound substrates^28^. When the concentration of selenium increased to 80,000 μg Se/L, a stress concentration, the gene of SULT1A, whose product is located in the sulfur and glucosinolates metabolism pathway, increased 4.05-fold in the roots (Table 4). This suggests that selenation also occurred, as selenium increased to a high concentration in the roots of *C. hupingshanensis* seedlings. The response of selenation to selenium is not the same in the leaves. Whether challenged with a low or high concentration of selenium, the expression of SULT1A increased 4.94 or 7.17-fold (Table 2). That is, the response of selenation was more common in leaves when the *C. hupingshanensis* seedlings were treated with selenium. Therefore, we can conclude that selenation is the more common method of selenium stress tolerance in *C. hupingshanensis* seedlings (Fig. 8).

### Degradation of selenoproteins is important for selenium detoxification

Both of the Selenomethionine and selenocysteine are seleno-amino acids which can be misincorporated into proteins in plants. Cysteine plays an important role in maintaining the structure and function of proteins, including those involved in catalysis, redox regulation, formation of disulfide bridges and metal binding. The substitution of cysteine with selenocysteine in nonspecific selenoproteins could create either a diselenide bridge or a mixed selenide–sulfide bridge (or selenosulfide bridge) with different properties, and a deformed protein could be formed^13^. A non-specific selenocysteine incorporated into selenoproteins in other situations and non-specific accumulation of selenomethionine proteins are not considered to be as deleterious as the more reactive selenocysteine, and malformed proteins can be formed^13^. The formation of deformed or malformed selenoproteins induced by chaperone-mediated processes and the proteolysis of irreparable proteins through the lysosome or the ubiquitin–proteasome pathway (UPP) can also occur. The mechanisms of preventing the formation of selenoproteins are related to elevated selenium tolerance in plants^79^. The mechanisms of preventing the formation of selenoproteins are associated with increased selenium tolerance in plants^4^. In this study, the genes of E3 ubiquitin-protein ligase MUL1 and RNF13 (treated with 100 μg Se/L) as well as of ubiquitin- conjugating enzyme E2 7 (treated with 80,000 μg Se/L) were up-regulated with 4.87, 5.55 and 2.82-fold in the roots, respectively. These changes are similar to the observations in *Chlamydomonas reinhardtii*, *Stanleya pinnata* and rice^4,79,80^. However, the puzzling change was that the gene of the 26S proteasome regulatory subunit T5 (Rpt5), an important subunit for assembly of the 26S proteasome, was down-regulated with 9.70-fold in all tissues and at both selenium concentrations. On the other hand, regarding phagosome and phagolysosome, the gene of cysteine-type peptidase was up-regulated with 9.98 fold in the roots (treated with 100 μg Se/L), and the gene of VHA-a2 was up-regulated with 10.24 fold in the leaves (treated with 80,000 μg Se/L). All these results indicate that the degradation of selenoproteins plays an important role in selenium detoxification (Figs 8 and 9A,B).

## Methods

### Plant materials

The seeds of *C. hupingshanensis* were harvested from the Yutangba Se mining field, which is located on the Enshi area in western Hubei province, China. Plants were grown in a growth chamber with light illumination (ca. 1600 mol^−2^ ms^−1^) over a 16/8 day and night at 20 ± 2 °C. Hoagland solution was sprayed every two days. Five-month-old *C. hupingshanensis* seedlings were harvested. The roots were washed first with tap water and then with deionized water (≥18 MΩ, Millipore, Bedford^TM^, USA) to exclude contamination from the surface. The washed seedlings, which were cultured in 100 mL of Hoagland solution, were divided into three experimental groups based on the results of our previous studies: control, supplementation with 100 μg Se L^−1^ and supplementation with 80,000 μg Se L^−1^, using sodium selenite (analytical reagent, Sinopharm Chemical Reagent Co., Ltd, Shanghai, China). After 24 hours, the *C. hupingshanensis* seedlings were harvested.

### RNA isolation, library preparation and sequencing

For the transcriptome sequencing, the roots and leaves were separated and analyzed independently. According to the manufacturer’s protocol, total RNA was extracted using a Qiagen total RNA isolation system (RNeasy Plant Mini Kit, 74904, Qiagen). The total RNA samples were treated by the following protocol: DNA degraded by DNase I; the oligo (dT) magnetic beads were used for mRNA enrichment; the mRNA was then fragmented into short fragments by mixing with the fragmentation buffer. Then, the cDNA was synthesized using PrimeScript™ Double Strand cDNA Synthesis Kit (Takara) according to the manufacturer’s protocol. The double-strand cDNA was purified with magnetic beads. And then 3′-end single nucleotide A (adenine) addition was performed. Finally, sequencing adaptors were ligated to the fragments. The fragments were enriched by PCR amplification. During the QC step, the Agilent 2100 Bioanalyzer and ABI StepOne Plus Real-Time PCR System were used to qualify and quantify the sample library. The primer information for the real-time quantitative PCR was shown in Table 5. Actin of *C. Hupingshanensis* (Chp Actin) served as internal controls to normalize the targets for quantification. The libraries were then sequenced on the Illumina HiSeq^TM^ 2000 platform.

**Table 5:** The information of primer for real-time quantitative PCR.

Gene name	Abbreviation	Primer	
ATP-binding cassette, subfamily B (MDR/TAP), member 1	ABCC1	P1	CGACCCACTATGTCCACTGTG
		P2	TGCCTTGTGTTTACGTCTGTTC
Adenylyl-sulfate reductase 1	APR1	P1	GCTCTTGAGAAATACGGAAACG
		P2	ACGGCAACACTCTTGATGACC
Glutaredoxin C-10	GrxC10	P1	TGTTGGATGGACTCCGACG
		P2	AAGACGATGCGAGGTTTACG
Thiol methyltransferase 2	SMT	P1	AGTTCTGTCAATTTCACCTACCAC
		P2	GGATAACGAACTTGCTCCAGATAC
Aryl sulfotransferase	SUL1A	P1	GGTTCCGGCATAAGTAGACAATC
		P2	CTCGCCACCATACCTAAATCC
Sulfate transporter 2.1	Sultr 2.1	P1	TGGCTGCTTGACTGTCCTG
		P2	GATTCGTTGTGGGAGAGGC
Sulfite oxidase	SOX	P1	GAAGAGGGACGGGAGTGATG
		P2	CGATTTCTCCAGCGACGAC
Thioredoxin 1	Trx 1	P1	CACTGAGCATCATTGCGTTATC
		P2	CGCCACTTCCTTGACTTCATC
Aspartate aminotransferase	AAT	P1	CAAAGTCTGTTGGTCGGGTG
		P2	TGAAAGCCGCCAATCCC
Cystathionine gamma-synthase	CγS	P1	TAAATGTCGTGGAACAGCGG
		P2	TCCTTACATAGCACCATCTTTCG
Cystathionine beta-lyase	CBL	P1	GCCACCATATACATCATCTCCAG
		P2	GCACTCCACTTTACCAAACAGC
5-methyltetrahydropteroyl- triglutamate-homocysteine methyltransferase	MET	P1	TTGTGGTTGGCAGGATTGG
		P2	TGGCGGGTCAGAAGGATG
Selenocysteine-lyase	SL	P1	TCTCTCAACTTGTCTATGTCTGGC
		P2	TGCTCGTTTCATCAATGCTTC
ATP-binding cassette, subfamily C (CFTR/MRP), member 2	ABCC2	P1	GTCCCAGATTCAAAGATAAACCG
		P2	GCAGGAGCAATAACAATAAGAGC
glutathione S-transferase 12	GST-12	P1	GAGTGTTTGGCGACAGTAGAAG
		P2	GACGGTTGGTATGTAAGGTTTG
glutathione S-transferase tau 4	GST-u4	P1	CCCTTTCAGTCGTAGAGTGGAG
		P2	ACATGGCTTTCTCGTAAGGATC
E3 ubiquitin-protein ligase	E3	P1	AGAGAGTAAGAGACGGTGTAGGATG
		P2	TCTAAGACGGTTGATACGACGAC
Cysteine protease	RD19A	P1	GGGAAATGAAGGTGAAGCAAG
		P2	ACAAATCGGACAACTCCCATC
ubiquitin-conjugating enzyme E2	E2	P1	GAACTCGTCTCTCTTCTCTCGC
		P2	GTCATTCCCGCTAAACTATCC
26 S proteasome regulatory subunit T5	RPT5	P1	CTAATGGCTCGTGCCTGTG
		P2	TGTCTCCGCTTACTTCACTGTC
V-type H+-transporting ATPase subunit I	VHAA2	P1	GGGAACAATGACCTGAACAAG
		P2	GTGGCAGTAACTACACGAGACG
pyridoxine 4-dehydrogenase	PLR1	P1	GCAGATGCTTCAGACAGACC
		P2	GCTCTCAAGGATGGTGTAAGG
tubulin beta	TUB	P1	AAACCAATCCTCTTCCCACTC
		P2	CCACTTCCCAGAACTTAGCAC
3′-phosphoadenosine 5′-phosphoselenate synthase	PAPSeS	P1	GGTATCTGGCATTGGTGGAG
		P2	CCGGTTAGAGGGTTATTGTCG
adenosine phosphoelenate	APSe	P1	TGGTTTCGATATTCCCGTCTC
		P2	ATCTTGCCTTGCGTCTTGTC

### Transcriptome assembly and annotation

The raw data were obtained after deep transcriptome sequencing. After quality analysis using the fastqc program (version 0.10.1), the raw data were processed to clip sequencing adapters and filter low-quality reads using Trimmomatic software (version 0.30). The remaining clean reads were used to assemble transcripts using the Trinity program (http://trinityrnaseq.sourceforge.net/) embedded with three individual modules (Inchworm, Chrysalis and Butterfly) which were run consecutively. The parameters for Trinity included the following: –seq-Type fq, –min_contig_length 100, –min_glue 3, –group_pairs_distance 250, –path_reinforcement_distance 85 and –min_kmer_cov 3. The following parameters were also used in Trinity: min_glue = 1, V = 10, edge-thr = 0.05, min_kmer_cov = 2, path_reinforcement_distance = 150, and group_pairs_distance = 500. The assembled contigs were finally joined together to make scaffolds. The Gene Indices Clustering Tools (TGICL, version 2.1) program was used to form unigenes, and Phrap (http://www.phrap.org/) was used to assemble the scaffolds and cluster them.

To annotate these unigenes, all sequences were subjected to blastx alignment (e-value < 1e-5) with the NR (non-redundant protein sequence database, release 20130408), Swiss-Prot (release 2013_03), KEGG (Kyoto Encyclopedia of Genes and Genomes) and COG (Clusters of Orthologous Groups) databases. The results from these alignments were used to determine the direction of these sequences. Lastly, the sequences that were not aligned to any database were subjected to a ESTScan analysis. For function annotation, a local Blast was used to search against the NT (NCBI nucleotide database), NR, Swiss-Prot, KEGG and COG databases. Blast hits from the NR database was then used to determine gene ontology (GO) terms of unigenes. The Blast2GO (http://www.blast2go.com/b2ghome) program was run to obtain the GO terms.

### Differential gene expression analysis

Expression profiling of unigenes was performed. The gene expression levels were quantified using the software package RNASeq by Expectation Maximization (RSEM). The fragments per kilobase per million reads (FPKM) method, which is able to eliminate the influence of different gene lengths and sequencing discrepancy on the calculation of gene expression, was used to estimate the expression level of each gene. Therefore, the gene expression levels normalized as FPKM values can be directly used to assess the differences in gene expression among samples. The FPKM value was calculated according to the length of each unigene and the number of reads mapped to the gene. Statistical analysis was performed to identify differentially expressed genes (DEGs). The false discovery rate (FDR) was calculated to adjust the p-value threshold in the expression analysis. If the FDR was small and the fold change was large, the difference in expression between the two samples was large. The criteria used to detect DEGs were FDR ≤ 0.001 and fold change (Se treated/not treated) ≥1 or ≤−1. In addition, the GO and KEGG pathway analyses were performed for the DEGs similar to the method described above.

## Electronic supplementary material

Supplementary Information
