# Supplementary material for: Comparative transcriptomics provides novel insights into the mechanisms of selenium tolerance in the hyperaccumulator plant Cardamine hupingshanensis
Source: Sci Rep. 2018 Feb 12;8:2789. doi: 10.1038/s41598-018-21268-2 (PMC5809607; doi:10.1038/s41598-018-21268-2)
Supplement: Supplementary file 1 — Supplementary Information [file 41598_2018_21268_MOESM1_ESM.doc]

**Comparative transcriptomics provides novel insights into the mechanisms of selenium tolerance** **in the hyperaccumulator plant *Cardamine hupingshanensis***

Yifeng Zhou^a,b,c^, Qiaoyu Tang^b^, Meiru Wu^b^, Di Mou^b^, Hui Liu^c^, Shouchuang Wang^a^, Chi Zhang^c^, Li Ding^c^, Jie Luo^a,*^

Emails and institutions addresses:

Yifeng Zhou (77416757@qq.com), Qiaoyu Tang (330375856@qq.com), Meiru Wu (1060198820@qq.com), Di Mou (772640410@qq.com), Hui Liu (905172468@qq.com), Shouchuang Wang (shouchuang.wang@webmail.hzau.edu.cn), Chi Zhang (1378060165@qq.com), Li Ding (1191998934@qq.com), Jie Luo (jie.luo@mail.hzau.edu.cn).

^a^ National Key Laboratory of Crop Genetic Improvement and National Center of Plant Gene Research (Wuhan), Huazhong Agricultural University, Wuhan 430070, China

^b^ Key Laboratory of Biological Resources Protection and Utilization of Hubei Province, Hubei University for Nationalities, Enshi 44500, China

^c^ Collage of Biological Science and Technology, Hubei University for Nationalities, Enshi 44500, China

Correspondence and requests for materials should be addressed to J. L(jie.luo@mail.hzau.edu.cn)

Telephone number: 86-27-87280113; Fax number: 86-27-87280113.

**Supplementary Information**

| No. | intercommunity | GeneID | log2Ratio | Up-Down Regulation | Probability | Blast nr |
| --- | --- | --- | --- | --- | --- | --- |
| 1 | mutual gene in four groups | c10770_g1_i1 | -6.35 | Down | 0.847 | BnaA04g17890D [Brassica napus] |
| 2 |  | c10770_g2_i1 | -8.69 | Down | 0.835 | probable 2-oxoacid dependent dioxygenase [Brassica rapa] |
| 3 |  | c20438_g1_i3 | -8.72 | Down | 0.838 | hypothetical protein CARUB_v10015992mg [Capsella rubella] |
| 4 |  | c26522_g1_i1 | -9.59 | Down | 0.910 | tubulin [Camelina sativa] |
| 1 | mutual gene in low and high selenium concentration groups in root | c37547_g1_i1 | 9.82 | Up | 0.923 | hypothetical protein CARUB_v10017775mg [Capsella rubella] |
| 2 |  | c32104_g1_i1 | 9.57 | Up | 0.908 | - |
| 3 |  | c23709_g1_i1 | 9.44 | Up | 0.899 | tubulin alpha-4 chain [Arabidopsis thaliana] |
| 4 |  | c22177_g1_i2 | 9.40 | Up | 0.897 | hypothetical protein EUTSA_v10008053mg [Eutrema salsugineum] |
| 5 |  | c21081_g1_i5 | 9.39 | Up | 0.896 | hypothetical protein CARUB_v10008291mg, partial [Capsella rubella] |
| 6 |  | c9203_g1_i1 | 9.31 | Up | 0.890 | - |
| 7 |  | c15152_g1_i2 | 8.77 | Up | 0.842 | hypothetical protein JCGZ_06007 [Jatropha curcas] |
| 8 |  | c21700_g1_i2 | 8.76 | Up | 0.877 | hypothetical protein CARUB_v10011940mg [Capsella rubella] |
| 9 |  | c23549_g1_i2 | 7.77 | Up | 0.942 | hypothetical protein ARALYDRAFT_475260 [Arabidopsis lyrata subsp. lyrata] |
| 10 |  | c19560_g1_i5 | 7.49 | Up | 0.859 | hypothetical protein AALP_AA7G239400 [Arabis alpina] |
| 11 |  | c41536_g1_i1 | 5.15 | Up | 0.843 | hypothetical protein AALP_AA1G140500 [Arabis alpina] |
| 12 |  | c11622_g1_i2 | 4.88 | Up | 0.832 | hypothetical protein AALP_AA1G340200 [Arabis alpina] |
| 13 |  | c4063_g1_i1 | 4.76 | Up | 0.803 | thioesterase family protein [Arabidopsis thaliana] |
| 14 |  | c12744_g2_i1 | 4.63 | Up | 0.820 | expressed protein [Arabidopsis lyrata subsp. lyrata] |
| 15 |  | c8451_g1_i1 | 4.61 | Up | 0.822 | hypothetical protein CARUB_v10000015mg [Capsella rubella] |
| 16 |  | c20025_g1_i2 | 4.25 | Up | 0.814 | hypothetical protein ARALYDRAFT_477490 [Arabidopsis lyrata subsp. lyrata] |
| 17 |  | c19580_g1_i1 | 4.21 | Up | 0.811 | uncharacterized protein [Arabidopsis thaliana] |
| 18 |  | c13131_g1_i1 | 4.16 | Up | 0.802 | - |
| 19 |  | c9795_g1_i1 | 4.06 | Up | 0.805 | hypothetical protein ARALYDRAFT_905664 [Arabidopsis lyrata subsp. lyrata] |
| 20 |  | c23603_g1_i2 | 4.02 | Up | 0.803 | hypothetical protein ARALYDRAFT_491537 [Arabidopsis lyrata subsp. lyrata] |
| 21 |  | c39257_g1_i1 | 4.00 | Up | 0.805 | - |
| 22 |  | c15094_g2_i1 | 3.84 | Up | 0.807 | hypothetical protein ARALYDRAFT_890890 [Arabidopsis lyrata subsp. lyrata] |
| 23 |  | c25587_g1_i1 | 3.67 | Up | 0.805 | hypothetical protein ARALYDRAFT_895665 [Arabidopsis lyrata subsp. lyrata] |
| 24 |  | c18259_g2_i4 | -6.37 | Down | 0.894 | hypothetical protein EMIHUDRAFT_79785 [Emiliania huxleyi CCMP1516] |
| 25 |  | c36512_g1_i1 | -7.57 | Down | 0.920 | defensin-like protein 205 [Arabidopsis thaliana] |
| 26 |  | c15278_g1_i1 | -8.40 | Down | 0.802 | hypothetical protein CARUB_v10014989mg [Capsella rubella] |
| 27 |  | c27268_g1_i1 | -8.40 | Down | 0.802 | hypothetical protein CARUB_v10019654mg [Capsella rubella] |
| 28 |  | c14524_g1_i1 | -8.42 | Down | 0.804 | - |
| 29 |  | c25273_g1_i4 | -8.43 | Down | 0.805 | RING/U-box domain-containing protein [Arabidopsis thaliana] |
| 30 |  | c14468_g3_i2 | -8.57 | Down | 0.821 | hypothetical protein H257_18843, partial [Aphanomyces astaci] |
| 31 |  | c25339_g4_i1 | -8.77 | Down | 0.842 | UDP-glucoronosyl/UDP-glucosyl transferase family protein [Arabidopsis lyrata subsp. lyrata] |
| 32 |  | c22996_g1_i3 | -8.89 | Down | 0.854 | hypothetical protein ARALYDRAFT_474095 [Arabidopsis lyrata subsp. lyrata] |
| 33 |  | c14584_g1_i1 | -8.89 | Down | 0.855 | hypothetical protein AALP_AA3G092900 [Arabis alpina] |
| 34 |  | c26791_g2_i2 | -8.99 | Down | 0.864 | - |
| 35 |  | c8277_g1_i2 | -9.04 | Down | 0.868 | receptor-like protein kinase [Arabidopsis thaliana] |
| 36 |  | c14986_g1_i2 | -9.08 | Down | 0.872 | hypothetical protein CARUB_v10013301mg [Capsella rubella] |
| 37 |  | c17114_g2_i1 | -9.21 | Down | 0.883 | - |
| 38 |  | c25282_g2_i4 | -9.52 | Down | 0.905 | hypothetical protein EUTSA_v10010597mg [Eutrema salsugineum] |
| 39 |  | c19954_g1_i4 | -9.92 | Down | 0.929 | endonuclease 2 [Arabidopsis thaliana] |
| 1 | mutual gene in low and high selenium concentration groups in leaves | c22186_g1_i5 | 11.68 | Up | 0.986 | hypothetical protein CARUB_v10020890mg [Capsella rubella] |
| 2 |  | c20629_g1_i2 | 10.33 | Up | 0.958 | hypothetical protein CARUB_v10017603mg [Capsella rubella] |
| 3 |  | c8460_g1_i1 | 10.22 | Up | 0.953 | - |
| 4 |  | c13442_g1_i2 | 9.78 | Up | 0.933 | hypothetical protein EUTSA_v10006305mg [Eutrema salsugineum] |
| 5 |  | c17903_g2_i3 | 9.73 | Up | 0.930 | BnaC05g22370D [Brassica napus] |
| 6 |  | c26422_g1_i5 | 9.28 | Up | 0.902 | hypothetical protein ARALYDRAFT_475431 [Arabidopsis lyrata subsp. lyrata] |
| 7 |  | c21926_g1_i2 | 9.26 | Up | 0.901 | hypothetical protein CARUB_v10007571mg [Capsella rubella] |
| 8 |  | c25188_g1_i3 | 9.25 | Up | 0.900 | hypothetical protein ARALYDRAFT_897659 [Arabidopsis lyrata subsp. lyrata] |
| 9 |  | c22163_g1_i1 | 9.11 | Up | 0.889 | hypothetical protein EUTSA_v10021302mg [Eutrema salsugineum] |
| 10 |  | c22144_g1_i6 | 8.97 | Up | 0.877 | PREDICTED: golgin subfamily A member 6-like protein 22 [Brassica rapa] |
| 11 |  | c24274_g1_i2 | 8.87 | Up | 0.868 | ATPAO2 [Arabidopsis lyrata subsp. lyrata] |
| 12 |  | c14919_g1_i2 | 8.78 | Up | 0.858 | hypothetical protein CARUB_v10027175mg [Capsella rubella] |
| 13 |  | c19052_g1_i1 | 8.63 | Up | 0.843 | uncharacterized protein [Arabidopsis thaliana] |
| 14 |  | c14005_g1_i1 | 8.60 | Up | 0.839 | - |
| 15 |  | c18036_g2_i4 | 8.46 | Up | 0.823 | hypothetical protein CARUB_v10001487mg [Capsella rubella] |
| 16 |  | c26436_g1_i1 | 6.71 | Up | 0.849 | BnaC05g41670D [Brassica napus] |
| 17 |  | c19266_g1_i9 | 6.30 | Up | 0.910 | putative glycine-rich RNA-binding protein [Chorispora bungeana] |
| 18 |  | c26649_g1_i3 | 5.26 | Up | 0.831 | multidrug resistance-associated protein 14 [Arabidopsis thaliana] |
| 19 |  | c24788_g1_i2 | 5.23 | Up | 0.841 | - |
| 20 |  | c24366_g2_i1 | 4.94 | Up | 0.826 | hypothetical protein CARUB_v10003509mg [Capsella rubella] |
| 21 |  | c15496_g1_i1 | 3.66 | Up | 0.807 | ATGOLS3 [Arabidopsis lyrata subsp. lyrata] |
| 22 |  | c33674_g1_i1 | -3.31 | Down | 0.801 | hypothetical protein MTR_029s0005 [Medicago truncatula] |
| 23 |  | c19001_g1_i2 | -3.50 | Down | 0.802 | hypothetical protein CARUB_v10001618mg [Capsella rubella] |
| 24 |  | c9285_g2_i1 | -4.07 | Down | 0.810 | - |
| 25 |  | c25527_g5_i3 | -5.98 | Down | 0.836 | BnaC03g42080D [Brassica napus] |
| 26 |  | c21981_g1_i3 | -8.38 | Down | 0.813 | hypothetical protein CARUB_v10018863mg [Capsella rubella] |
| 27 |  | c14666_g1_i2 | -8.39 | Down | 0.815 | ankyrin repeat family protein [Arabidopsis lyrata subsp. lyrata] |
| 28 |  | c15323_g1_i2 | -8.46 | Down | 0.824 | - |
| 29 |  | c24658_g1_i1 | -8.49 | Down | 0.826 | hypothetical protein ARALYDRAFT_323872 [Arabidopsis lyrata subsp. lyrata] |
| 30 |  | c20581_g2_i4 | -8.50 | Down | 0.827 | Endoribonuclease/protein kinase IRE1-like protein [Arabidopsis thaliana] |
| 31 |  | c18282_g1_i1 | -8.59 | Down | 0.838 | monofunctional riboflavin biosynthesis protein RIBA 3 [Arabidopsis thaliana] |
| 32 |  | c23987_g1_i4 | -8.60 | Down | 0.839 | WD-40 repeat family protein [Arabidopsis lyrata subsp. lyrata] |
| 33 |  | c25632_g1_i3 | -8.69 | Down | 0.849 | uncharacterized protein [Arabidopsis thaliana] |
| 34 |  | c9130_g2_i2 | -8.72 | Down | 0.852 | BnaA10g00540D [Brassica napus] |
| 35 |  | c25630_g1_i1 | -8.76 | Down | 0.856 | hypothetical protein EUTSA_v10007078mg [Eutrema salsugineum] |
| 36 |  | c18057_g1_i3 | -8.80 | Down | 0.861 | hypothetical protein EUTSA_v10017082mg [Eutrema salsugineum] |
| 37 |  | c25527_g5_i1 | -8.82 | Down | 0.863 | hypothetical protein EUTSA_v10020016mg [Eutrema salsugineum] |
| 38 |  | c18955_g1_i2 | -8.94 | Down | 0.875 | hypothetical protein ARALYDRAFT_494519 [Arabidopsis lyrata subsp. lyrata] |
| 39 |  | c8580_g1_i1 | -9.01 | Down | 0.880 | - |
| 40 |  | c23230_g1_i2 | -9.04 | Down | 0.883 | hypothetical protein ARALYDRAFT_900392 [Arabidopsis lyrata subsp. lyrata] |
| 41 |  | c15257_g1_i1 | -9.05 | Down | 0.884 | - |
| 42 |  | c21981_g1_i2 | -9.45 | Down | 0.914 | hypothetical protein CARUB_v10018863mg [Capsella rubella] |
| 43 |  | c19221_g1_i3 | -9.64 | Down | 0.925 | hypothetical protein EUTSA_v10001571mg [Eutrema salsugineum] |
| 44 |  | c19683_g2_i3 | -9.71 | Down | 0.929 | late embryogenesis abundant protein (LEA) family protein [Arabidopsis thaliana] |
| 45 |  | c22186_g1_i6 | -10.17 | Down | 0.951 | expressed protein [Arabidopsis thaliana] |
| 46 |  | c15335_g1_i2 | -10.59 | Down | 0.965 | hypothetical protein CARUB_v10014585mg, partial [Capsella rubella] |
| 1 | mutual gene in root and leaves low selenium concentration groups | c17493_g1_i2 | 9.00 | Up | 0.864 | BnaC08g29100D [Brassica napus] |
| 2 |  | c25306_g2_i1 | 8.74 | Up | 0.839 | Ras-related protein RABA1c [Arabidopsis thaliana] |
| 3 |  | c25630_g1_i1 | 8.68 | Up | 0.833 | hypothetical protein EUTSA_v10007078mg [Eutrema salsugineum] |
| 4 |  | c11058_g2_i1 | -8.90 | Down | 0.856 | hypothetical protein CARUB_v10006352mg [Capsella rubella] |
| 5 |  | c17687_g1_i2 | -9.97 | Down | 0.931 | - |
| 1 | mutual gene in root and leaves high selenium concentration groups | c19241_g2_i1 | 10.03 | Up | 0.942 | - |
| 2 |  | c23230_g1_i2 | 9.09 | Up | 0.885 | hypothetical protein ARALYDRAFT_900392 [Arabidopsis lyrata subsp. lyrata] |
| 3 |  | c24366_g2_i1 | 4.05 | Up | 0.819 | hypothetical protein CARUB_v10003509mg [Capsella rubella] |
| 4 |  | c18605_g1_i1 | 3.59 | Up | 0.809 | hypothetical protein CARUB_v10016497mg [Capsella rubella] |
| 5 |  | c26773_g2_i1 | -7.99 | Down | 0.811 | hypothetical protein CARUB_v10012798mg [Capsella rubella] |
| 6 |  | c10442_g1_i1 | -8.63 | Down | 0.843 | hypothetical protein PHAVU_011G120500g [Phaseolus vulgaris] |
| 7 |  | c19052_g1_i1 | -9.13 | Down | 0.888 | uncharacterized protein [Arabidopsis thaliana] |
| 8 |  | c17114_g2_i1 | -9.21 | Down | 0.895 | - |

**Supplementary Table S1. Differentially expressed genes (DEGs) of *C. hupingshanensis***
